# Rotational motion of Left Ventricle by Harmonic Phase on radial tagging

**DOI:** 10.1186/1532-429X-18-S1-P332

**Published:** 2016-01-27

**Authors:** Shokoufeh Golshani, Nafiseh Babaee, Abbas Nasiraei-Moghaddam

**Affiliations:** grid.411368.90000000406116995Amirkabir University of Technology, Tehran, Iran (the Islamic Republic of)

## Background

The rotational motion of the Left Ventricle (LV) is a valuable measure in assessment of cardiac mechanics and can be estimated best through radial tagging. For practical quantitative application of this newly developed sequence, corresponding analyzing tools need to be developed. In this study, we presented a processing method based on peak-combination Harmonic Phase (HARP) approach to obtain displacement fields in polar coordinate.

## Methods

**Figure 1:**
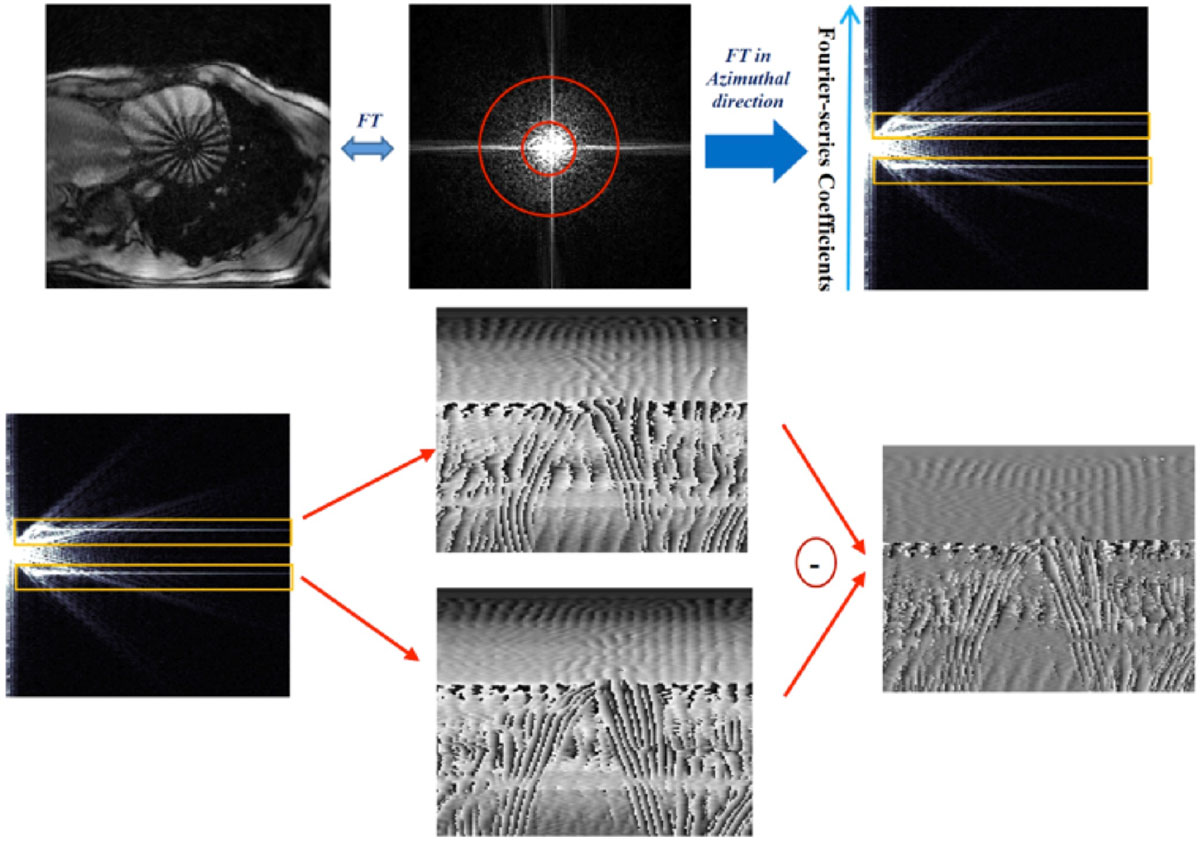
**The beginning of the PFT procedure is shown on the k-space of the first cardiac frame**. After performing the first 1D-FFT of the radial tagged images, the tagging energy is concentrated in two highlighted lines, which are the reminiscent of spectral peaks in the 1-1 SPAMM. Inspired by HARP, only few Fourier-coefficients around one of the highlighted lines can be isolated to form a 2D complex image of the tagging fundamental frequency. This was repeated for the conjugate highlighted line and therefore, the conjugate harmonic phase was reconstructed. Based on the peak-combination HARP, the two images were subtracted to improve the accuracy of the resulting phase map. The harmonic phase images were then unwrapped and used for calculation of the displacement field.

Short-axis human heart images with radial tagging pattern were acquired using a radial k-space sampling scheme. The data consisted of 19 cardiac frames obtained in a single breath-hold of 12 seconds duration. We exploited a Polar Fourier Transform for the reconstruction and processing of polar images. The method is based on two angularly Fourier Transforms separated by a Hankel-transform in the radial direction. Figure 1 illustrates the k-space of the first cardiac frame. After performing the first 1D-FFT of the radial tagged images, the tagging energy is concentrated in two highlighted lines which are the reminiscent of spectral peaks in the 1-1 SPAMM. Inspired by HARP, only few Fourier-coefficients around one of the highlighted lines can be isolated to form a 2D complex image of the tagging fundamental frequency. This was repeated for the conjugate highlighted line and therefore, the conjugate harmonic phase was reconstructed. Based on the peak-combination HARP, the two images were subtracted to improve the accuracy of the resulting phase map. The harmonic phase images were then unwrapped and used for calculation of the displacement field.

## Results

**Figure 2:**
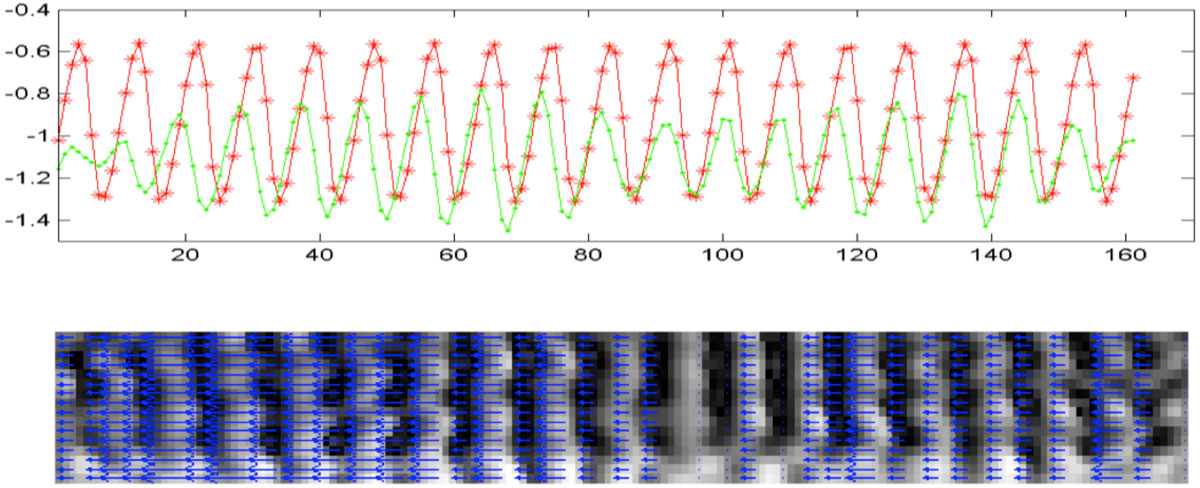
**The overall phase changes across the myocardium for frames one and six are plotted in top row**. Comparing the two phase plots, results in the angular displacement of the LV at each area, as illustrated in bottom row.

The outcome of this procedure for a sample radial tagging image, which has been transferred to polar coordinate system during PFT, is shown in the second row of figure 1. The area with regular periodic phase change corresponds to myocardium region. The overall phase changes across the myocardium for frames one and six are plotted in Figure 2, top part. Comparing the two phase plots, results in the angular displacement of the LV at each area, as illustrated in figure 2, bottom panel.

## Conclusions

HARP method for 1-1 SPAMM has a dual version in the Radial tagging in which the spectral peaks are replaced with the highlighted lines after the first angular FFT. Therefore, the same advantages one may expect from HARP in conventional tagging is transferred to radial tagging, if we use PFT in image reconstruction. It should be further noted that the rotational motion can be directly measured in the polar rather than Cartesian coordinates.

